# Cat Flea Coinfection with *Rickettsia felis* and *Rickettsia typhi*

**DOI:** 10.1089/vbz.2023.0122

**Published:** 2024-04-08

**Authors:** Hanna J. Laukaitis-Yousey, Kevin R. Macaluso

**Affiliations:** ^1^ Department of Microbiology and Immunology, Frederick P. Whiddon College of Medicine, University of South Alabama, Mobile, Alabama, USA.; ^2^ Department of Microbiology and Immunology, University of Maryland School of Medicine, Baltimore, Maryland, USA.

**Keywords:** *Rickettsia*, transmission, flea feces, flea-borne disease

## Abstract

**Purpose:**

Flea-borne rickettsioses, collectively referred to as a term for etiological agents *Rickettsia felis*, *Rickettsia typhi*, and RFLOs (*R. felis*-like organisms), has become a public health concern around the world, specifically in the United States. Due to a shared arthropod vector (the cat flea) and clinical signs, discriminating between *Rickettsia* species has proven difficult. While the effects of microbial coinfections in the vector can result in antagonistic or synergistic interrelationships, subsequently altering potential human exposure and disease, the impact of bacterial interactions within flea populations remains poorly defined.

**Methods:**

In this study, *in vitro* and *in vivo* systems were utilized to assess rickettsial interactions in arthropods.

**Results:**

Coinfection of both *R. felis* and *R. typhi* within a tick-derived cell line indicated that the two species could infect the same cell, but distinct growth kinetics led to reduced *R. felis* growth over time, regardless of infection order. Sequential flea coinfections revealed the vector could acquire both *Rickettsia* spp. and sustain coinfection for up to 2 weeks, but rickettsial loads in coinfected fleas and feces were altered during coinfection.

**Conclusion:**

Altered rickettsial loads during coinfection suggest *R. felis* and *R. typhi* interactions may enhance the transmission potential of either agent. Thus, this study provides a functional foundation to disentangle transmission events propelled by complex interspecies relationships during vector coinfections.

## Introduction

Rickettsial infections, which are spread by blood-feeding arthropods, are both historically and currently important diseases affecting human health worldwide. In the United States, flea-borne rickettsioses, collectively caused by *Rickettsia typhi* (murine typhus) and *Rickettsia felis* (flea-borne spotted fever), have become a concern in California, Texas, and Hawaii, where murine typhus has reemerged as an endemic febrile illness (Azad et al., 1997; Blanton et al., 2015; Ruiz et al., 2020). Cases have more than doubled in the last decade in southern California and Texas alone (Blanton, 2019; Blanton and Walker, 2017; Blanton et al., 2015), where it is a reportable disease by the public health department (Anstead, 2020; CA.gov, 2023) and has recently been associated with human deaths (Alarcon et al., 2023).

However, both flea-borne diseases are undistinguishable febrile illnesses, making diagnoses complicated. In addition, emerging flea-borne rickettsial species, such as *R. felis*-like organisms (RFLOs), are increasingly detected among flea populations around the globe with pathogenicity to vertebrate hosts inconclusive (Maina et al., 2018; Maina et al., 2016; Tay et al., 2015). With the ubiquitous detection of *R. felis* and RFLOs within fleas across the United States, why cases of murine typhus are re-appearing in endemic regions remains unresolved.

The cosmopolitan distribution of murine typhus cases is perpetuated by the biology of the transmitting vector, the cat flea, *Ctenocephalides felis.* Cat fleas are known to harbor several bacterial pathogens (*e.g.*, *R. felis*, *R. typhi*, and *Bartonella henselae*) and are the most prevalent flea species found on peri-domestic and companion animals (cats and dogs) (Mullins et al., 2018; Rust, 2017). As infected fleas feed on a host, transmission can occur not only through salivary secretions but also through the inoculation of infected flea feces into abrasions in host skin or mucosal membranes (Gillespie et al., 2009), providing increased opportunity for pathogen dispersal. The diversity and implications of sympatric flea-associated *Rickettsia* spp. have only recently begun to be appreciated, leaving little understood regarding their geographic distribution and potential as human or animal pathogens.

Multiple factors influence the spread of vector-borne diseases, including host susceptibility to infection, microbiome, vectorial capacity, environment, and, most importantly, the tripartite host-vector-pathogen interactions. Within vector populations, coinfections with multiple pathogens are known to occur and can alter disease epidemiology, resulting in antagonistic, mutualistic, or synergistic interactions between organisms (Ginsberg, 2008; Goertz et al., 2017; Levin and Fish, 2001; Levin et al., 2018). For example, the relationship governing tick-borne rickettsial coinfections suggests a primary infection excludes transovarial transmission of a secondarily acquired organism from infected females to an egg clutch (Burgdorfer et al., 1980; Macaluso et al., 2002).

For flea-borne rickettsiae, naturally, *R. felis-*infected fleas acquire *R. typhi* at a lower prevalence, suggesting an antagonistic relationship may exist (Noden et al., 1998). However, interpretations of the impact of coinfection on the epidemiology of vector-borne diseases are highly stringent on laboratory settings, bacterial strains, and vector species (Levin et al., 2018). Although carriage of multiple rickettsial agents by fleas occurs in nature (Eremeeva et al., 2008; Maina et al., 2016), the influence of the temporal effects of flea-borne rickettsial interactions is not well understood.

While *R. felis* and *R. typhi* are known to colonize their rat flea and cat flea hosts for life without an observable effect on vector viability (Adams et al., 1990; Farhang-Azad and Traub, 1985; Farhang-Azad et al., 1984; Healy et al., 2017; Wedincamp and Foil, 2002), the epidemiology surrounding cases of flea-borne rickettsioses is confounding as field studies seldom report coinfections (Eremeeva et al., 2008; Maina et al., 2016) (Table 1). Therefore, it is hypothesized that if rickettsial interactions influence pathogen persistence in flea populations, then infection order will impact rickettsial maintenance by fleas.

**Table 1.** Detection of Flea-Borne Rickettsiae in the United States

Rickettsial species detected	Geographic location	Flea species	Pooled/individual	Total fleas tested	Rickettsia positive	Flea pools tested	Prevalence (%)*^a^*	MIR (%)*^b^*
*Rickettsia felis*	California	Mixed fleas	Pooled^c^	2352	463	1271	—	19.7
*Rickettsia typhi*	Los Angeles County, California	*Ctenocephalides felis*	Individual^d^	106	18	106	16.98	—
		*C. felis*	Pooled^e^	2144	454	1146	—	21.2
		*Echidnophaga gallinacea*	Pooled^e^	17	4	9	—	23.5
		*Pulex irritans*	Pooled^e^	3	1	3	—	33.3
		*Xenopsylla cheopis*	Pooled^e^	5	2	4	—	40
		*X. cheopis*	Pooled^f^	233	43	119	—	18.5
	Orange County, California	*C. felis*	Individual^g^	597	169	597	28.31	—
		*C. felis*	Pooled^e^	1405	428	727	—	30.5
		*P. irritans*	Pooled^e^	109	9	55	—	8.3
	Riverside County, California	*C. felis*	Individual^h^	55	11	55	20.00	—
		*C. felis*	Pooled^h^	14	1	1	—	7.1
	San Bernardino County, California	*C. felis*	Individual^i^	1258	28	118	2.23	—
		*C. felis*	Pooled^i^	1258	152	570	—	12.1
	Oahu, Hawaii	*X. cheopis*	Individual^j^	367	104	367	28.34	—
	Oklahoma	*C. felis*	Pooled^k^	90	29	47	—	32.2
	Galveston, Texas	*C. felis*	Pooled^l^	57	4	19	—	7
	East Texas	*C. felis or P. irritans*	Individual^m^	12	2	12	16.67	—
		*C. felis or P. irritans*	Pooled^m^	184	19	28	—	10.3
	California	Mixed fleas	Pooled^c^	2352	17	1271	—	0.72
	Los Angeles County, California	*C. felis*	Pooled^e^	2144	18	1146	—	0.84
		*X. cheopis*	Pooled^f^	233	30	119	—	12.9
	Orange County, California	*C. felis*	Individual^g^	597	8	597	1.34	—
*R. typhi*	Orange County, California	*C. felis*	Pooled^e^	1405	7	727	—	0.5
		*P. irritans*	Pooled^e^	109	4	55	—	3.7
	Oahu, Hawaii	*X. cheopis*	Individual^j^	367	8	367	2.18	—
	Oklahoma	*C. felis*	Pooled^k^	90	3	47	—	3.3
	Galveston, Texas	*C. felis*	Pooled^l^	57	1	19	—	1.8
		*C. felis*	Pooled^n^	333	13	63	—	3.9
*R. felis+R. typhi*	California	Mixed fleas	Pooled^c^	2352	21	1271	—	0.89
	Los Angeles County, California	*C. felis*	Pooled^e^	2144	10	1146	—	0.47
		*E. gallinacea*	Pooled^e^	17	1	9	—	5.9
		*X. cheopis*	Pooled^f^	233	7	119	—	3.0
	Orange County, California	*C. felis*	Pooled^e^	1405	22	727	—	1.6
	Oahu, Hawaii	*X. cheopis*	Individual^j^	367	2	367	0.54	—
	Oklahoma	*C. felis*	Pooled^k^	90	3	47	—	3.3
*R. felis-*like	Orange County, California	*C. felis*	Individual^g^	597	212	597	35.51	—
	Galveston, Texas	*C. felis*	Pooled^n^	333	11	63	—	3.3
*R. typhi+R. felis-*like	Galveston, Texas	*C. felis*	Pooled^n^	333	3	63	—	0.9
*Rickettsia* genus	Southeastern Georgia	*C. felis*	Pooled^o^	1805	120	622	—	6.7

^a^ Prevalence = ([number of individual *Rickettsia*-positive fleas/total fleas tested] × 100).

^b^ MIR = ([number of *Rickettsia*-positive pools/total fleas tested] × 100).

^c^ Karpathy et al. (2009); ^d^Nelson et al. (2018); ^e^Eremeeva et al. (2012); ^f^Abramowicz et al. (2011); ^g^Maina et al. (2016); ^h^Mullins et al. (2018); ^i^Abramowicz et al. (2012); ^j^Eremeeva et al. (2008); ^k^Noden et al. (2017); ^l^Blanton et al. (2019); ^m^Wang et al. (2022); ^n^Blanton et al. (2016); ^o^Brown et al. (2022).

MIR, minimum infection rate.

In this study, an *in vitro* and *in vivo* model of *R. felis* and *R. typhi* coinfection was used to assess interaction phenotypes. While distinct interspecies growth profiles were observed in both systems, coinfection of individual arthropod cells was visualized by microscopy using species-specific antibodies. Similarly, naive fleas acquired both *Rickettsia* spp. and sustained coinfection for 2 weeks. A combination of both an *in vitro* and flea infection bioassay provides a multidimensional platform to examine the complex biology perpetuating sympatric distributions of rickettsial species.

## Materials and Methods

### Fleas, cell lines, and *Rickettsia*

Cat fleas were purchased from Elward II Laboratory (Soquel, CA) and maintained using an artificial dog system (Wade and Georgi, 1988). Before use in each bioassay, a subset of fleas was confirmed to be pathogen-specific (*R. felis* and *R. typhi*) free by quantitative PCR (qPCR) (Danchenko et al., 2021; Laukaitis et al., 2022).

For *in vitro* infection bioassays, the *Ixodes scapularis*-derived cell line (ISE6), and the rickettsial isolate, *R. felis* strain LSU passage 3 was maintained as previously described (Brown et al., 2015; Danchenko et al., 2021; Laukaitis et al., 2022; Pornwiroon et al., 2006). *R. typhi* strain Wilmington passage <5 was originally cultured in Vero cells using Dulbecco's Modified Eagle Media (DMEM) supplemented with 5% fetal bovine serum (FBS) at 34°C with 5% CO_2_. It was purified from Vero cells through needle lysis for *in vitro* coinfection assays in ISE6 cells. For flea infections, *R. typhi* was grown in ISE6 cells. Rickettsial infection was monitored by Diff-Quik staining (Brown et al., 2015; Danchenko et al., 2021; Laukaitis et al., 2022; Pornwiroon et al., 2006).

### *In vitro* coinfection assays

While the lack of a currently available flea cell line limits the ability to directly assess flea-specific infection mechanisms, other arthropod cells, such as tick-derived cell lines, have successfully facilitated the study of obligate intracellular organisms in the Rickettsiales order (Felsheim et al., 2006; Kurtti et al., 2016; Wang et al., 2020a; Wang et al., 2020b). Specifically, *R. felis* strains have been isolated from insects using ISE6 cells (Pornwiroon et al., 2006; Thepparit et al., 2011). Therefore, ISE6 cells were seeded into 48-well plates at a density of 5 × 10^5^ cells/well or seeded onto glass coverslips in 24-well plates at a density of 8 × 10^5^ cells/well to enumerate rickettsiae by qPCR or visualize rickettsial infection by microscopy, respectively. All plates were incubated at 32°C with 5% CO_2_ for 48 h before infection.

For coinfections, each *Rickettsia* sp. was enumerated by *Bac*Light viability stain kit (Sunyakumthorn et al., 2008) to determine a multiplicity of infection (MOI) of 5 rickettsiae/cell. Coinfections consisted of simultaneous and sequential inoculation with reciprocal primary infections. To inoculate ISE6 cells, rickettsiae were partially purified as previously established (Simser et al., 2001; Sunyakumthorn et al., 2008). Briefly, infected cells were lysed using a 27-gauge needle. To remove large host cell debris, the inoculum was centrifuged at 275 *g* for 10 min. The supernatant was filtered through a 2.0 μm filter and rickettsiae were pelleted at 16,200 *g* for 10 min and resuspended in the appropriate volume of media.

For sequential coinfections, the partially purified primary *Rickettsia* sp. was added to a monolayer of ISE6 cells at a low volume, and host cell contact was induced by centrifugation at 300 *g* for 5 min. Plates were incubated for 1 h at 32°C, after which unbound bacteria were removed by pipetting, and the secondary *Rickettsia* sp. was added to the cells in the same manner. Growth curve analyses were initiated after removing unbound rickettsiae from the secondary inoculation. Whole well contents were collected by washing with 1 × phosphate buffered saline (PBS) beginning at 12 h postinfection (hpi) to 1, 3, 5, and 7 days postinfection (dpi).

Contents were transferred to 1.7 mL microcentrifuge tubes and pelleted at 16,200 *g* for 10 min, in which media were then removed before genomic DNA (gDNA) extraction. Rickettsial growth was calculated as a change over time determined by enumeration of genomic equivalents through a species-specific qPCR. To compare the effects of coinfection on species-specific growth, parallel assays of single infections were carried out. Cells grown on coverslips were collected at representative time points (12 h, 1 day, and 5 days) for immunofluorescence staining. All *in vitro* assays were performed in duplicate for qPCR and microscopy analysis.

### Immunofluorescence

Cells grown on coverslips were washed thrice with 1 × PBS and fixed using 4% paraformaldehyde, permeabilized with 0.5% Triton-X100, and blocked with 3% bovine serum albumin (BSA). A polyclonal rabbit anti-*Rickettsia* I1789 antibody (provided by Ted Hackstadt; National Institutes of Health's Rocky Mountain Laboratories) diluted 1:1000 was used to probe for *R. felis*, while specific detection of *R. typhi* was achieved using a monoclonal mouse antibody directed against *R. typhi* OmpB (*Rt-*mOmpB; provided by Lee Fuller; Fuller labs) at a dilution of 1:2000.

Primary antibodies were detected using conjugated secondary antibodies, Alexa Fluor 488 goat anti-rabbit (A11008; 1:1000 dilution; Invitrogen) and Alexa Fluor 594 goat anti-mouse (A11005; 1:1000 dilution; Invitrogen). Host cell actin was stained using Alexa Fluor Plus 647 Phalloidin (A30107; 1:1000 dilution; Invitrogen). Coverslips were mounted with VECTASHEILD^®^ Hard Set™ antifade mounting medium with DAPI (4′,6-diamidino-2-phenylindole; Vector Laboratories, Inc.) for nuclear staining. Secondary antibody-only controls were included as negative controls for nonspecific binding. Samples were visualized using a confocal Nikon A1 microscope (S10RR027535).

### Flea coinfection

For flea infection, both *Rickettsia* spp. were grown independently in ISE6 cells, and rickettsiae were enumerated using the *Bac*Light kit. To assess coinfection in the vector, cages were prepared with ∼200 mixed-sex cat fleas and prefed heat-inactivated bovine blood (HemoStat Laboratories) for 24 h. Cat fleas were then starved for 6 h before exposure to a *Rickettsia*-infected bloodmeal at an infectious dose of 3 × 10^10^ rickettsiae (Brown et al., 2015; Danchenko et al., 2021).

**FIG. 1.**
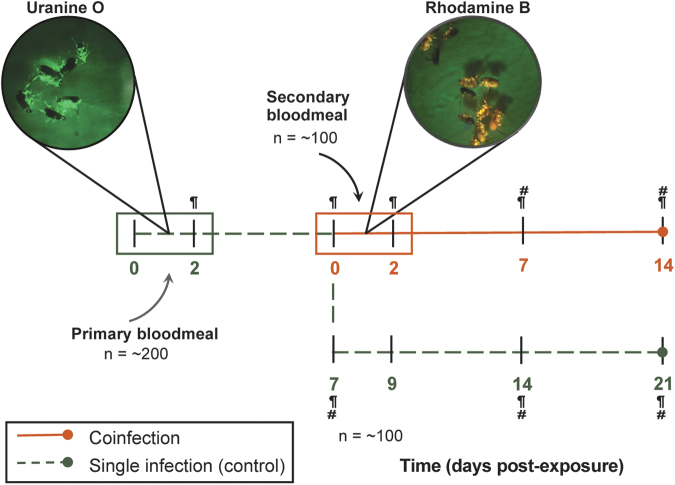
Experimental design for flea coinfections. Primary *Rickettsia*-infected bloodmeals (*green box*) were supplemented with the fluorescent biomarker, uranine O, and fleas were allowed continuous access for 48 h. Fleas were maintained on uninfected defibrinated blood until 7 dpe, in which a subset of fleas (*orange line*) were exposed to a secondary *Rickettsia*-infected bloodmeal with rhodamine B, RhoB (*orange box*). The remaining subset of fleas (*dashed green line*) were maintained on uninfected blood for the duration of the study to serve as a control for a single rickettsial infection. Individual fleas (five male and five female) and feces were collected, indicated by *paragraph* and *hashtag symbols*, respectively, for detection of rickettsiae by qPCR. dpe, Days postexposure; qPCR, quantitative PCR.

The primary infectious bloodmeal was supplemented with the fluorescent biomarker uranine O at a concentration of 0.05 mg/L, and fleas were allowed continuous access for 48 h (Mascari and Foil, 2010). Postfeeding, fleas were assessed under a fluorescence stereo microscope (NIGHTSEA; Electron Microscopy Sciences) for the presence of uranine O, and only fluorescent fleas were returned to the cage and maintained on uninfected, defibrinated blood (Fig. 1). Five days following the removal of the primary infectious bloodmeal, the initial flea cage was split into two cohorts. The first cohort (∼100 fleas) remained a control infection (primary *Rickettsia* sp. alone).

The other cohort (∼100 fleas) was exposed to a secondary *Rickettsia*-infected bloodmeal supplemented with the fluorescent biomarker rhodamine B at a concentration of 0.025 mg/L for 48 h (Hirunkanokpun et al., 2011; Mascari and Foil, 2010). Again, fluorescence was assessed to confirm bloodmeal acquisition. Reciprocal primary infections were completed in parallel for a total of three independent replicates (Fig. 1). Coinfections were monitored over 2 weeks. A subset of fleas (5 male and 5 female) was collected initially postexposure and weekly thereafter (30 fleas total per time point), along with flea feces. Flea feces were collected from flea cages after removal of flea carcasses, larvae, and eggs. Rickettsial prevalence and load within individual fleas and fecal samples were analyzed using a species-specific qPCR assay (Table 2).

**Table 2.** Primers and Probes Used for Species-Specific Quantitative PCR

Oligo name: primer set (5*′*-3′); probe (5′-3′)	Sequence	Citation
Rt557F	TGGTATTACTGCTCAACAAGCT	Henry et al. (2007)
Rt678R	CAGTAAAGTCTATTGATCCTACACC	
Rt4446_4476.Cy5	Cy5/TAATAGCAGCACCAGCATTAACTTTTGAAAC	This study
Rfel.OmpB.FOR	TAATTTTAACGGAACAGACGGT	Odhiambo et al. (2014)
Rfel.OmpB.REV	GCCTAAACTTCCTGTAACATTAAAG	
Rfel.OmpB.HEX/FAM	HEX/or FAM/TGCTGCTGGTGGCGGTGC	
18srRNA.FOR	GAGTTCCGACCAGAGATGGA	This study
18srRNA.REV	CGCAGAAACTACCATCGACA	
18srRNA.FAM	FAM/TGCCTTGCTCACCGTTTGACTTGGTG	
ISE6.cal.FOR	AGCAGGGAACTTTCAAGCTG	Harris et al. (2018)
ISE6.cal.REV	AGAAAGGCTCGAACTTGGTG	
ISE6.cal.HEX	HEX/AGACCTCTGAAGATGCCCGCTTT	

### DNA extraction and qPCR

Fleas were surface sterilized (Danchenko et al., 2021; Laukaitis et al., 2022) and homogenized using 2- and 3-mm stainless steel beads in a Bead Ruptor 96 (Omni International). Flea lysates were incubated overnight at 56°C, and gDNA was extracted from the sample using the DNeasy Blood and Tissue Kit (Qiagen) following the manufacturer's blood extraction protocol. Flea feces were collected and 200 μL of 1 × PBS with 20 μL of Proteinase K was added to each microcentrifuge tube. Feces were incubated overnight at room temperature and gDNA was extracted following the same blood extraction protocol used for flea lysates. For host cells, cell pellets collected in 1.7 mL microcentrifuge tubes were prepared following the manufacturer's instructions for cultured cells.

Rickettsial and host gene copies were quantified by qPCR with the appropriate primers and probes (Table 2) using iTaq Universal Probes Supermix (Bio-Rad) on a LightCycler 480 II (Roche Life Sciences). Standard curves were generated by creating 10-fold serial dilutions of pCR4-TOPO plasmids containing the *R. felis ompB*, *R. typhi ompB*, *C. felis 18sRNA*, or ISE6 *calreticulin* genes to quantify each target sequence. Amplification conditions were as follows: An initial denaturation step at 95°C for 3 min, followed by 45 cycles of denaturation at 95°C for 15 s, annealing, and elongation at 60°C for 60 s with fluorescence acquisition in single mode.

### Statistical analyses

For assessment of rickettsial growth between species during *in vitro* and *in vivo* (flea and feces) single infections at a given time point, an unpaired *t*-test with Welch's correction for unequal variances was performed when appropriate. If data did not meet normal distribution assumptions, a Mann-Whitney U test was used. To compare growth kinetics *in vitro* during coinfection to single infections over time, a two-way analysis of variance (ANOVA) was performed with Dunnett's multiple comparisons test. *R. felis* data were log transformed for normality. Rickettsial prevalence between conditions was compared using Fisher exact test. Due to the highly non-normal distribution of mean rickettsial loads for coinfected fleas, a computationally intensive, but assumption-free randomization test, also referred to as the permutation test, was applied (Edgington and Onghena, 2007).

To examine whether the mean distribution varied between coinfection and single infections, a resampling method involving 1000 permutations was performed for each analysis. The randomization test was carried out using Microsoft Excel. For changes in rickettsial loads in fleas and feces during coinfection, the number of rickettsiae detected during coinfection was divided by the average load detected during single infection, and statistical significance was determined by two-way ANOVA. Data were log2 transformed for graphical representation. To assess relationships between rickettsial loads detected in coinfected fleas, correlation analysis was performed. Other than the randomization test, all statistical analyses were performed using Prism 10 software (GraphPad Software Version 10.0.2). A *p* value <0.05 was considered statistically significant.

## Results

### Rickettsial growth kinetics in ISE6 cells during single infection

**FIG. 2.**
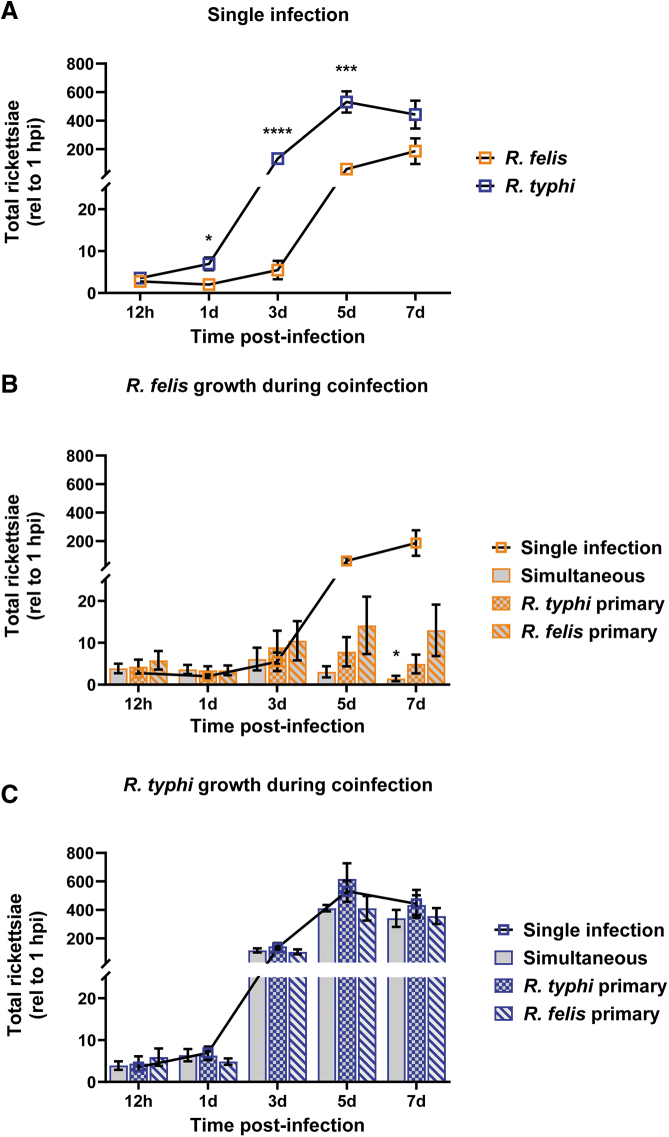
*In vitro* coinfection growth kinetics. ISE6 cells were infected at an MOI of 5 rickettsiae/cell. Whole well contents were collected beginning at 12 hpi and every other day thereafter for 1 week. **(A)** Representative growth curve kinetics of *Rickettsia felis* (*orange*) and *Rickettsia typhi* (*blue*) during single infections. An unpaired *t*-test with Welch's correction for unequal variances or Mann-Whitney U test was performed to determine significance between the rickettsial loads at a given time point. **(B)** Growth of *R. felis* during coinfection compared to single infection alone (superimposed line graph). **(C)** *R. typhi* growth during coinfections compared to single infection alone (superimposed line graph). All data are normalized to input bacteria at 1 h post-coinfection. Data are representative of mean ± SEM from two experiments, each with three technical replicates. Significance was assessed at a 95% confidence interval (*p < 0.05; ***p < 0.001; ****p < 0.0001) by two-way ANOVA with Dunnett's multiple-comparison test to assess variation in the means during coinfection compared to the control infection of the same species over time. Two-way ANOVA was performed on log-transformed data for *R. felis* infection. ANOVA, analysis of variance; hpi, hours postinfection; MOI, multiplicity of infection; SEM, standard error of the mean.

Tick cells, which served as a surrogate arthropod infection model, were exposed to rickettsiae, and growth was monitored over 7 days. Comparing interspecies growth kinetics, *R. typhi* displayed a shortened lag phase (0–24 hpi), reaching stationary phase at a faster rate (5 dpi) compared to *R. felis* (7 dpi) under the conditions examined (Fig. 2A). Furthermore, *R. typhi* reached significantly higher rickettsial densities than *R. felis* beginning at 1 dpi.

### Impact of coinfection on rickettsial growth in ISE6 cells

To determine the effect of coinfection on rickettsial growth, simultaneous and sequential coinfections with reciprocal primary *Rickettsia* sp. were performed. Growth curves from each coinfection experiment were compared to single infections alone to analyze the influence of rickettsial interaction on normal growth kinetics of each species. During coinfection, *R. felis* exhibited limitations in the ability to reach an equivalent exponential growth phase when compared to single infection beginning at 5 dpi (Fig. 2B). By 7 dpi, *R. felis* loads were significantly decreased during simultaneous coinfection. Conversely, *R. typhi* growth was unaffected by the presence of *R. felis* during coinfection (Fig. 2C).

### Detection of rickettsiae within individual ISE6 cells

**FIG. 3.**
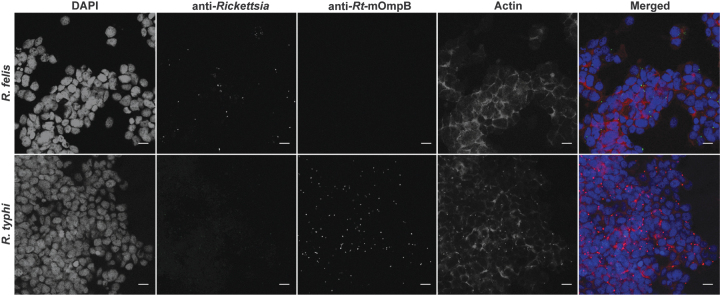
Antibody specificity of flea-borne rickettsiae. Fluorescence microscopy representing antibody specificity of anti-*Rickettsia* (*panel* 2) and anti-*Rt-*mOmpB (*panel* 3) antibodies during single species infection. *Rickettsia felis* (*green*) and *Rickettsia typhi* (*red*), host cell nuclei (*blue*), and actin (*magenta*). Images are representative of two independent experiments. Scale bar = 10 μm. *Rt-*mOmpB = *R. typhi* monoclonal outer membrane protein B antibody.

**FIG. 4.**
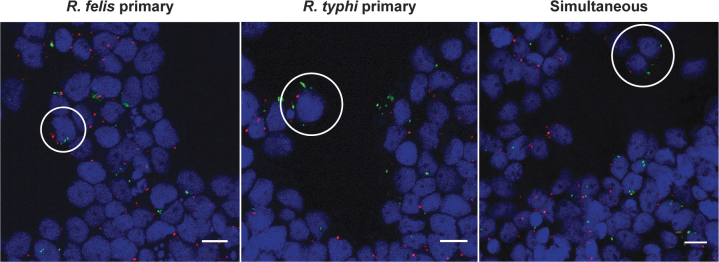
Assessment of rickettsial coinfection at 12 h in ISE6 cells by microscopy. Infected host cells were washed, and bound rickettsiae were differentially stained for *Rickettsia felis* (*green*) and *Rickettsia typhi* (*red*) during reciprocal sequential or simultaneous coinfection along with host cell nuclei (*blue*). Coinfected cells are designated by *white circles*. Images are representative of two independent replicates. Scale bar = 10 μm.

To determine if flea-borne rickettsiae can infect and occupy the same arthropod cell, tick cells were serially and simultaneously coinfected and visualized by fluorescence microscopy. The use of a monoclonal antibody generated against *R. typhi* was vital in allowing its distinction from that of *R. felis* (Fig. 3). At early time points post-coinfection, both *Rickettsia* spp. were visualized in individual cells under all experimental conditions (Fig. 4). However, cells visualized after 24 h of coinfection skewed host cell infection in favor of *R. typhi* (Supplementary Fig. S1), which was consistent with species-specific detection by qPCR.

### Flea infection kinetics of rickettsiae during single infection

**FIG. 5.**
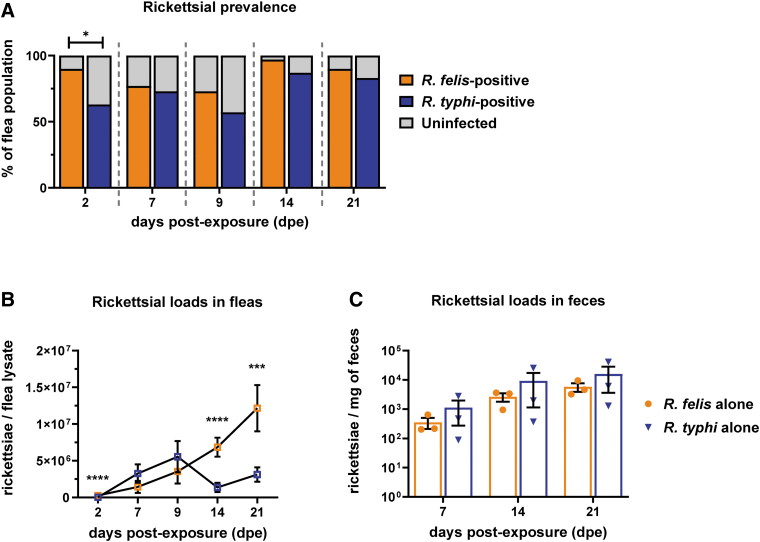
Single rickettsial species infection in cat fleas. Fleas were exposed to *Rickettsia felis* or *Rickettsia typhi*, independently, at an infectious dose of 3 × 10^10^ rickettsiae for 48 h. Individual fleas (five male and five female) were collected weekly over a 21-day period for enumeration of rickettsiae through species-specific qPCR. **(A)** Rickettsial prevalence, or the number of fleas positive for *Rickettsia felis* (*orange*) or *R. typhi* (*blue*) or negative (*gray*) out of the total number of fleas collected totaling 100%. **(B)** Rickettsial infection loads for *R. felis* (*orange*) and *R. typhi* (*blue*) over time in individual fleas. **(C)** Flea feces were collected weekly for 3 weeks, where *R. felis* (*orange*) and *R. typhi* (*blue*) loads were quantified by qPCR. For all data, significance was assessed at a 95% confidence interval with **(A)** Fisher exact test; **(B)** Mann-Whitney U test; **(C)** unpaired *t*-test with a Welch's correction for unequal variances (*p < 0.05; ***p < 0.001; ****p < 0.0001). Data represent mean ± SEM with 3 independent experiments for a total of 30 fleas.

Fleas were independently exposed to 3 × 10^10^ rickettsiae through an infectious bloodmeal to assess species-specific infection dynamics. Rickettsial prevalence (percentage of *Rickettsia*-positive fleas/total fleas collected) between species was not significantly different (Fig. 5A). However, infection dynamics varied in a species-specific manner. Increasing *R. felis* loads were observed weekly over a 21-day infection period, while *R. typhi* presented a more dynamic infection cycle in which significantly lower rickettsial loads were observed after 9 days postexposure (dpe) (Fig. 5B). As insect-borne rickettsiae utilize infectious feces as a mechanism of transmission to vertebrate hosts (Laukaitis and Macaluso, 2021), flea feces were collected weekly and assessed for rickettsial burden by qPCR. While an increase in rickettsiae over a 3-week period of infection was observed for both species (Fig. 5C), loads did not significantly differ at any time point examined.

### Impact of flea coinfection on rickettsial growth kinetics

**FIG. 6.**
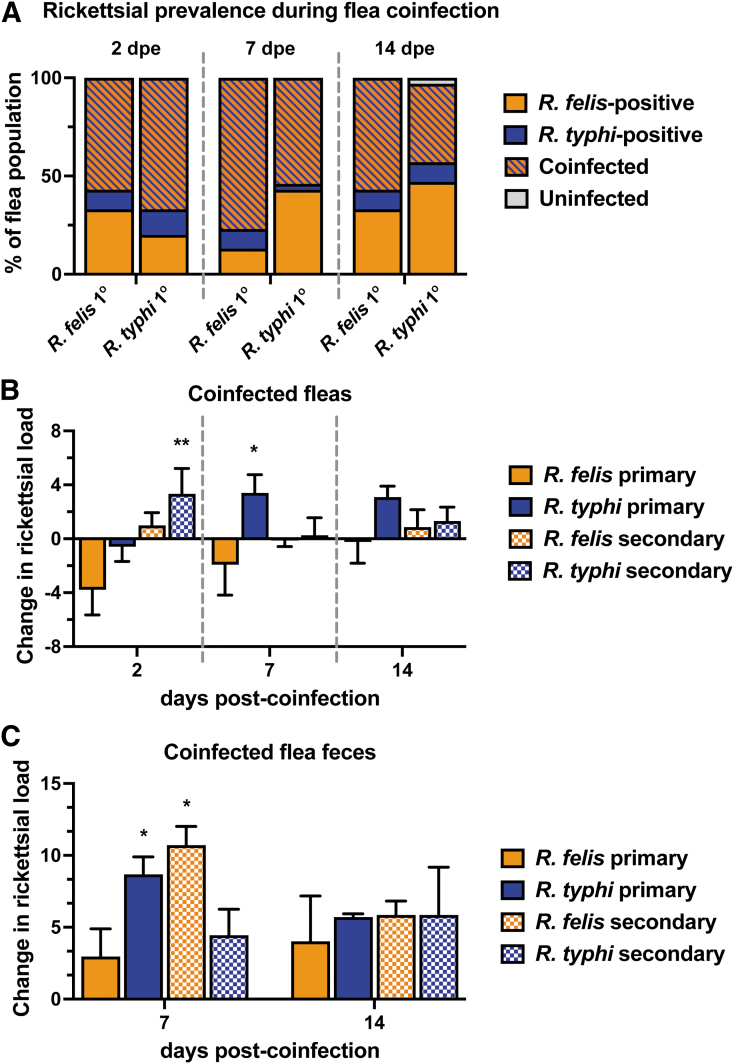
Rickettsial infection kinetics during cat flea coinfection. Fleas were exposed to sequential *Rickettsia*-infected bloodmeals at an infectious dose of 3 × 10^10^ rickettsiae for 48 h. Individual fleas (five male and five female) were assessed for the presence of rickettsiae by a species-specific qPCR assay. **(A)** Number of fleas that tested positive for *Rickettsia felis* only (*orange*), *Rickettsia typhi* only (*blue*), both species (*striped pattern*), or uninfected (*gray*) out of the total number of fleas collected totaling 100%. **(B)** Change in rickettsial load in coinfected fleas was calculated as a change from the single infection of the same species at a given time point. Data were log2 transformed for graphical representation. *R. felis* (*orange*) and *R. typhi* (*blue*) were detected when exposed as the primary bloodmeal (*solid bar*) or secondary bloodmeal (*checkered pattern*). **(C)** Flea feces were collected weekly for the detection of rickettsiae. Data were calculated as a change from the single infection of the same species at a given time point, and log2 transformed for graphical representation. *R. felis* (*orange*) and *R. typhi* (*blue*) were detected when exposed as the primary bloodmeal (*solid bar*) or secondary bloodmeal (*checkered pattern*). For all data, significance was assessed at a 95% confidence interval with **(A)** Fisher exact test; **(B)** randomization test with 1000 permutations; **(C)** two-way ANOVA with a Dunnett's *post-hoc* test (*p < 0.05; **p < 0.01). All data are representative of the mean ± SEM from 3 independent experiments for a total of 30 fleas.

To determine the ability of naive fleas to acquire both *R. felis* and *R. typhi*, fleas were exposed to sequential *Rickettsia*-infected bloodmeals. Sequential coinfections occurred 7 dpe to the primary infection, permitting rickettsiae to establish infection within the flea (Azad, 1990; Ito et al., 1975; Thepparit et al., 2013). Reciprocal coinfections were performed in parallel to determine if a priority effect upon pathogen acquisition occurred. Coinfection prevalence and rickettsial loads were monitored over a 14-day period, where fleas were collected weekly for rickettsial enumeration through qPCR using a species-specific assay. Fleas were able to maintain coinfection to similar percentages between treatments ranging from 55% at 2 dpe, 75% at 7 dpe, to 45% at 14 dpe (Fig. 6A).

To analyze the effects of coinfection on the growth kinetics of each *Rickettsia* sp. during flea infection, change differences were calculated based on single infection profiles. Coinfection led to significant differences in rickettsial loads during the 2-week period (Fig. 6B). *R. felis*-infected fleas acquired significantly higher *R. typhi* loads after removal of the bloodmeal, and *R. typhi* remained elevated 14 days later. In addition, *R. typhi* loads significantly increased 7 days after acquiring *R. felis*, which continued for the duration of the experiment.

Conversely, levels of *R. felis* declined after the acquisition of *R. typhi* within the first 2 weeks of coinfection when compared to single infection. *R. typhi*-infected fleas developed moderately higher loads of *R. felis* when *R. felis* was acquired as a secondary bloodmeal. Altogether, *R. typhi* loads generally increased in the presence of *R. felis* compared to single infections alone, where inverse effects were observed for *R. felis*. To determine the potential impacts of coinfection on transmission, rickettsial loads were monitored in excreted feces. Both *Rickettsia* spp. were detected at higher loads in feces when compared to single infection across all coinfection scenarios (Fig. 6C). Specifically, acquiring *R. felis* as a secondary exposure significantly propelled *R. typhi* and *R. felis* excretion in feces at 7 days post-coinfection.

### Correlation between rickettsial loads during flea coinfection

To examine whether changes in rickettsial loads during coinfection were driven by interspecies relationships, rickettsial loads detected for each species were graphed and re-analyzed for correlational significance. When fleas were pre-exposed to *R. felis*, detection of both species significantly trended in a positive direction with an *R*^2^ value of 0.73 at 2 days post-coinfection, 0.38 at 7 days post-coinfection, and 0.35 at 14 days post-coinfection (Supplementary Fig. S2A–C). However, there was no statistical evidence linking correlation between rickettsial loads when *R. typhi* was the primary exposure (Supplementary Fig. S2D–F).

## Discussion

The eco-epidemiological factors contributing to the reemergence of murine typhus in the United States is complicated by the circulation of several closely related flea-borne rickettsial agents, such as *R. typhi*, *R. felis*, and RFLOs (Abramowicz et al., 2012; Eremeeva et al., 2020; Eremeeva et al., 2012; Eremeeva et al., 2008; Karpathy et al., 2009; Maina et al., 2016; Williams et al., 1992). Overlapping peridomestic transmission cycles, where the cat flea is highly prevalent, present a likely scenario for bacterial interactions within the vector to occur. However, most field studies compile pooled flea sample data to assess rickettsial prevalence; thus, the true etiological agent and detection of coinfection remain difficult to define. Therefore, the goal of this study was to provide insight into the epidemiology surrounding flea-borne rickettsioses. The data presented herein suggest that *R. felis* and *R. typhi* coinfection prompts significant changes in rickettsial infection dynamics.

Consistent with studies in other arthropod cells, *R. felis* displayed a lag in growth lasting 3 days (Horta et al., 2006; Luce-Fedrow et al., 2014). Comparatively, *R. typhi* is primarily cultivated in vertebrate cell lines, such as Vero, macrophages, and L929 cells (Dreher-Lesnick et al., 2008; Rennoll-Bankert et al., 2015; Teysseire et al., 1992; Voss et al., 2020). Successful propagation of *R. typhi* in tick-derived cells, mimicking infection kinetics observed during vertebrate cell culture (Radulovic et al., 2002; Weiss et al., 1972), presents an opportunity to investigate arthropod-associated interrelationships. While ISE6 cells were investigated in this study because of their universal role in the isolation and propagation of *R. felis*, the use of other arthropod cell lines, such as *Aedes-*derived (C6/36, or AeAl2) or *Drosophila*-derived (S2) cells, would be of particular interest in future studies.

Sympatric distribution of rickettsiae fosters bacterial interactions to occur within communal transmitting vectors. Although the distinction of closely related rickettsial species has proven difficult, adaptation of a species-specific probe-based qPCR assay (Henry et al., 2007; Odhiambo et al., 2014), as well as polyclonal and monoclonal antibodies against *R. felis* and *R. typhi*, respectively, enabled discrimination within individual host cells.

In the presence of *R. typhi*, *R. felis* growth was limited, beginning at 5 dpi, regardless of infection order, suggesting that the accelerated growth of *R. typhi* consequently deprived *R. felis* of essential molecules required for growth. Tick-borne bacterial interactions describe competition between more closely related species (Cull et al., 2022; de la Fuente et al., 2002). Although the long-term persistence of cellular coinfection was not assessed, future studies examining intracellular niches during coinfection are warranted to fully encapsulate the impact carriage of two species has regarding resource allocation, host cell response, and sustainability.

Flea-borne rickettsiae are known to utilize multiple routes of transmission, including both vertical (to flea progeny) and horizontal (inoculation of infectious feces or salivary secretions to vertebrates) (Laukaitis and Macaluso, 2021). If imbibed in a bloodmeal, rickettsiae are exposed to the flea's midgut, which is regarded as the primary site of infection. Throughout infection, *R. typhi* remains largely centralized to the flea's digestive tract. As *R. typhi* reaches exponentially high levels of infection, it causes rupture of midgut epithelial cells, facilitating excretion through flea feces (Azad, 1990; Ito et al., 1975). This phenomenon was mirrored by the dynamic infection profile of *R. typhi* where rickettsial loads decline between 9 and 14 dpe.

Alternatively, *R. felis* can quickly disseminate to distal tissues from the midgut (*e.g*., the hindgut, reproductive tissues, and salivary glands) as early as 24 hpi (Danchenko et al., 2021; Laukaitis et al., 2022; Thepparit et al., 2013). Although also detected in flea feces, in similar levels to that of *R. typhi*, *R. felis* exhibited increased loads within its flea host over a 21-day period (Hirunkanokpun et al., 2011; Laukaitis et al., 2022; Reif et al., 2011; Reif et al., 2008; Thepparit et al., 2013), suggesting bacterial replication in other tissues compensate for those rickettsiae expelled in feces. The contrasting infection phenotypes presented in this study pose an intriguing opportunity to investigate the implications of distinct interspecies interactions between *Rickettsia* spp. and their flea host on transmission.

Interactions between rickettsiae have been examined, primarily in the context of tick-borne spotted fever *Rickettsia*, yet less is known in respect to insect-borne *Rickettsia*. A wild-caught *R. felis-*infected flea colony has been previously used to examine the relationship between *R. felis* and *R. typhi* during vector infection (Noden et al., 1998). However, wild-caught hematophagous arthropods, such as fleas and ticks, have difficulty adapting to artificial blood-feeding systems, presenting limitations in controlling rickettsial dose and, therefore, monitoring lifelong infection kinetics due to low vector success. Thus, the goal of this study was to assess rickettsial acquisition and sustainability in naive, *Rickettsia*-free fleas to control for these variables. Nonetheless, constitutively infected fleas present an opportunity to employ vertebrate host systems where natural horizontal and vertical transmission events can be simulated.

Sequential exposure to *Rickettsia*-infected bloodmeals using an artificial host system, revealed naive fleas were able to acquire both *Rickettsia* spp. and maintain coinfection for at least 2 weeks. Overall, independent of infection order, *R. typhi* loads were enhanced during coinfection compared to single infection alone, which was contrasting to the generalized trends observed for *R. felis*. However, by 14 days post-coinfection, both *R. felis* and *R. typhi* were detected at higher levels compared to singular infections, signifying fleas may tolerate overall higher rickettsial burdens during coinfection.

These data, complemented by strong correlational evidence, indicate prior exposure to *R. felis* provides an advantageous flea environment in which both species benefit. Importantly, higher detection within individual fleas may have implications on transmission efficacy through excreted flea feces. Although direct transmission to vertebrate hosts was not assessed in this study, enhanced detection of both *R. felis* and *R. typhi* in flea feces across all coinfection scenarios suggests coinfection may drive horizontal transmission. Use of a vertebrate host in future studies will elucidate whether higher rickettsial loads during coinfection enhance vertebrate exposure to either species.

Surveillance studies seldom detect flea-borne rickettsial coinfections, implying additional biological factors may influence microbial interactions in fleas, such as microbiome composition and vertebrate reservoir hosts. For example, stable vertical maintenance of *R. felis* by fleas impacts the richness of flea microbiota, but the consequence of dual rickettsial exposure remains elusive (Pornwiroon et al., 2007). Although in its infancy, flea genotyping through metagenomics studies has emphasized the circulation of distinct geographically displaced flea groupings throughout the world, harboring different microbiome compositions, therefore influencing pathogen dispersal (Manvell et al., 2022; Vasconcelos et al., 2018).

Interpretations of laboratory coinfections related to the epidemiology of vector-borne diseases are difficult as they only encapsulate a singular scenario and are highly stringent on strains used (Levin et al., 2018). Nonetheless, the results presented signify a situation in which *R. felis* and *R. typhi* interact within their vector host, resulting in exacerbated infection and excretion in flea feces. The confounding epidemiology surrounding the resurgence of murine typhus affiliated with the overlapping geographic distribution of rickettsiae stresses the importance of studying the implications of bacterial interactions. Further investigation into the contribution of coinfection on flea-borne rickettsial transmission, considering different flea strains, flea microbiomes, and timing of rickettsial exposure, is essential.

The study presented herein provides novel insight into rickettsial interactions within an arthropod environment. While distinct growth phenotypes exist between *Rickettsia* spp. and strains in mammalian backgrounds (McGinn and Lamason, 2021), direct comparison of kinetics during flea infection is limited. Coinfection of single cells and antagonistic growth phenotypes were observed during *in vitro* culture, but further investigation is warranted to uncover the molecular interplay occurring during dual infection. The enhanced rickettsial loads observed during flea coinfection present an exciting opportunity to investigate whether horizontal transmission events result in higher transmission rates to vertebrate hosts. In summary, this study provides insight into the interplay between closely related flea-borne rickettsiae, suggesting coinfection perpetuates bacterial success in the vector. Integration of *in vitro* and *in vivo* models can deconvolute interspecies interactions, providing a platform for greater understanding of factors governing flea-borne rickettsiosis epidemiology.

## Data Availability Statement

All relevant data are within the article and its Supplementary Figures S1 and S2.

## Supplementary Material

Supplemental data

Supplemental data
