# Supplementary figures and images for: Cat Flea Coinfection with Rickettsia felis and Rickettsia typhi
Source: Vector Borne Zoonotic Dis. 2024 Apr 8;24(4):201–13. doi: 10.1089/vbz.2023.0122 (PMC11035851; doi:10.1089/vbz.2023.0122)

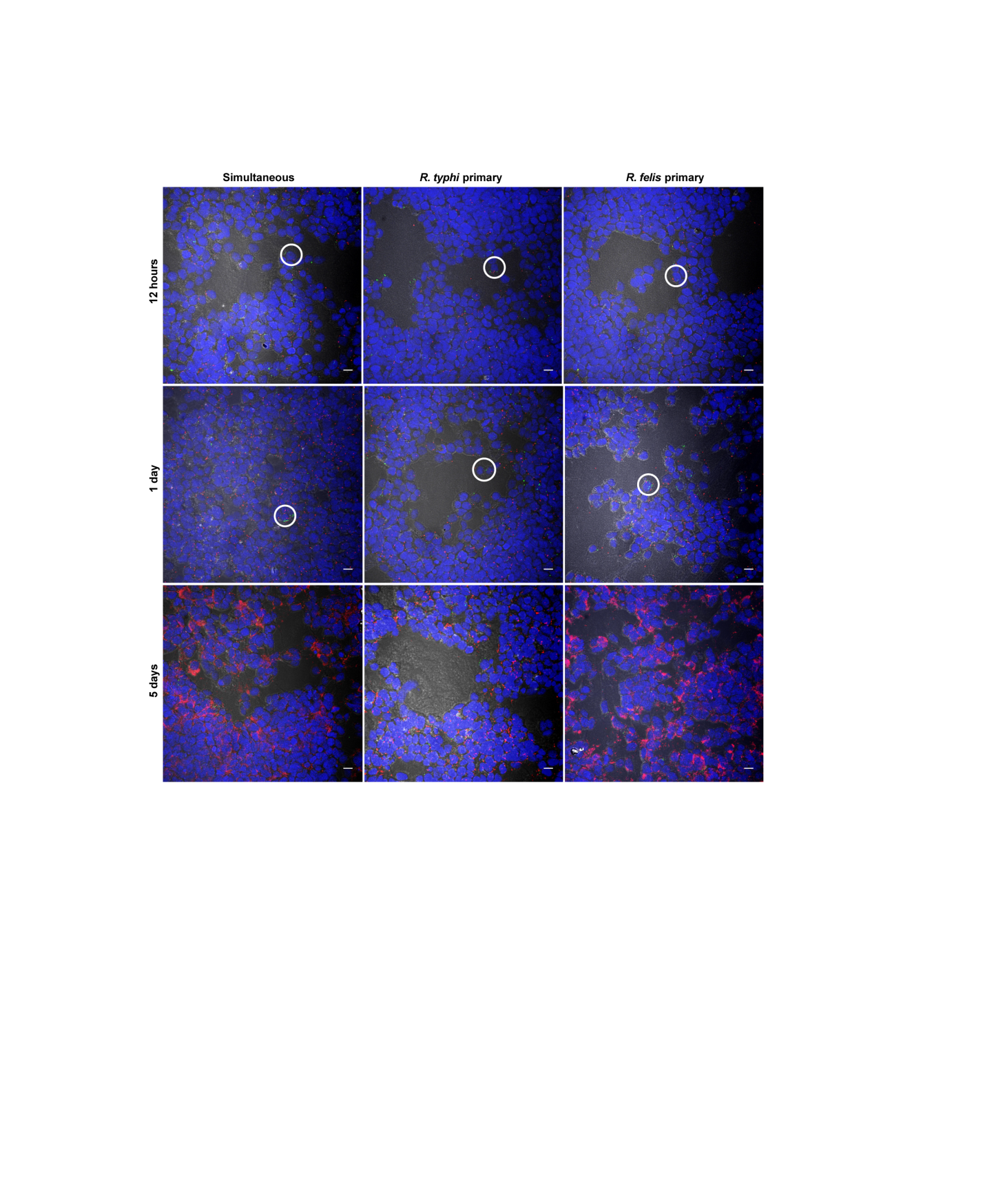

Supplement: Supplemental data [file Suppl_FigS1.docx]

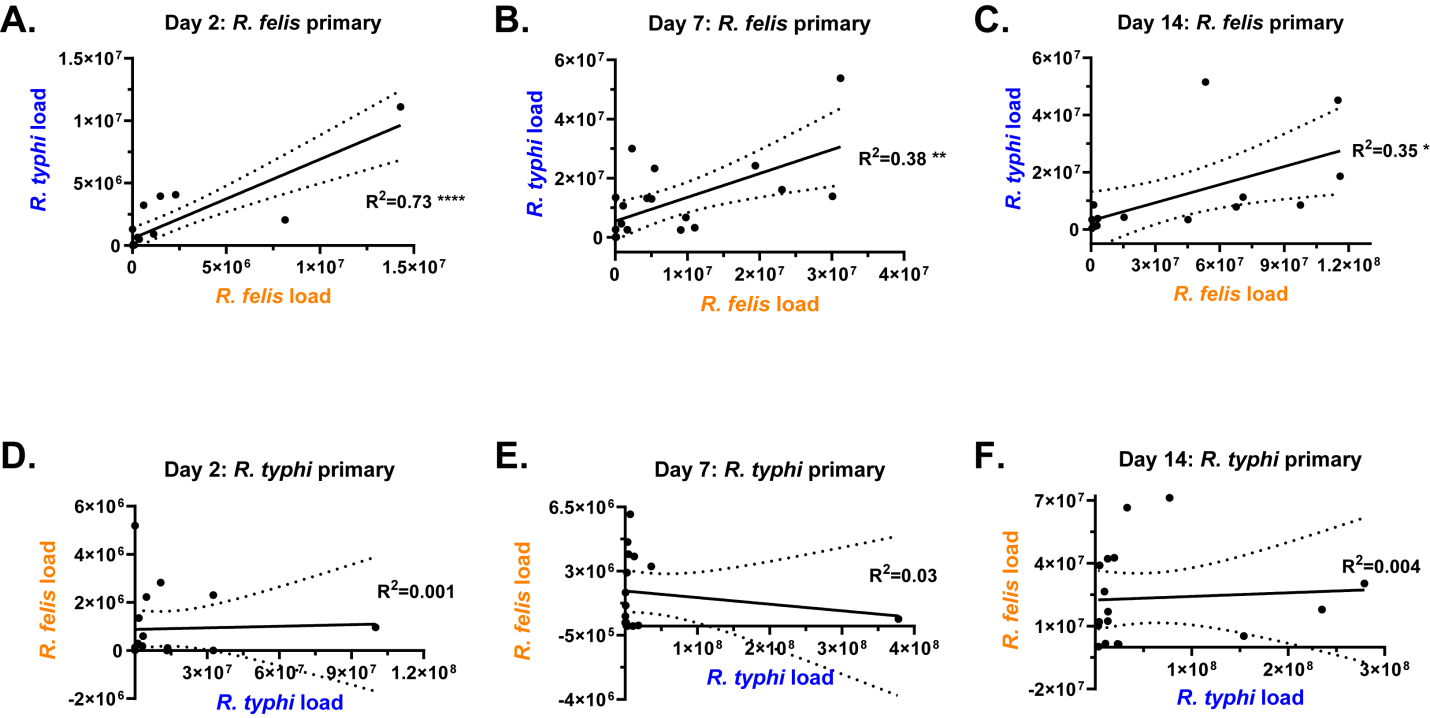

Supplement: Supplemental data [file Suppl_FigS2.docx]
